# Psychological symptoms, mental fatigue and behavioural adherence after 72 continuous days of strict lockdown during the COVID-19 pandemic in Argentina

**DOI:** 10.1192/bjo.2021.1065

**Published:** 2021-12-10

**Authors:** Fernando Torrente, Adrian Yoris, Daniel Low, Pablo Lopez, Pedro Bekinschtein, Gustavo H. Vázquez, Facundo Manes, Marcelo Cetkovich

**Affiliations:** Institute of Neuroscience and Public Policy, INECO Foundation, Argentina; and Institute of Cognitive and Translational Neurosciences, National Council of Science and Technology, INECO Foundation and Favaloro University, Argentina; Institute of Neuroscience and Public Policy, INECO Foundation, Argentina; and Institute of Cognitive and Translational Neurosciences, National Council of Science and Technology, INECO Foundation and Favaloro University, Argentina; Program in Speech and Hearing Bioscience and Technology, Harvard Medical School and MIT, USA; Institute of Neuroscience and Public Policy, INECO Foundation, Argentina; and Institute of Cognitive and Translational Neurosciences, National Council of Science and Technology, INECO Foundation and Favaloro University, Argentina; Institute of Neuroscience and Public Policy, INECO Foundation, Argentina; and Institute of Cognitive and Translational Neurosciences, National Council of Science and Technology, INECO Foundation and Favaloro University, Argentina; Department of Psychiatry, Queen's University Medical School, Canada; Institute of Neuroscience and Public Policy, INECO Foundation, Argentina; and Institute of Cognitive and Translational Neurosciences, National Council of Science and Technology, INECO Foundation and Favaloro University, Argentina; Institute of Neuroscience and Public Policy, INECO Foundation, Argentina; and Institute of Cognitive and Translational Neurosciences, National Council of Science and Technology, INECO Foundation and Favaloro University, Argentina

**Keywords:** Low- and middle-income countries, community mental health, behavioural fatigue, lockdown, Fatigue Assessment Scale

## Abstract

**Background:**

An early and prolonged lockdown was adopted in Argentina during the first wave of COVID-19. Early reports evidenced elevated psychological symptoms.

**Aims:**

To explore if the prolonged lockdown was associated with elevated anxiety and depressive symptoms; if mental fatigue was associated with lockdown adherence (a phenomenon called ‘behavioural fatigue’); and if financial concerns were associated with lockdown adherence and emotional symptoms.

**Method:**

The survey included standardised questionnaires to assess depressive (PHQ-9) and anxious (GAD-7) symptoms, mental fatigue, risk perception, lockdown adherence, financial concerns, daily stress, loneliness, intolerance to uncertainty, negative repetitive thinking and cognitive problems. LASSO regression analyses were carried out to predict depression, anxiety and lockdown adherence

**Results:**

The survey reached 3617 adults (85.2% female) from all provinces of Argentina after 72 days of lockdown. Data were collected between 21 May 2020 and 4 June 2020. In that period, Argentina had an Oxford stringency index of 85/100. Of those surveyed, 45.6% and 27% met the cut-offs for depression and anxiety, respectively. Mental fatigue, cognitive failures and financial concerns were correlated with psychological symptoms, but not with adherence to lockdown. In regression models, mental fatigue, cognitive failures and loneliness were the most important variables to predict depression, intolerance to uncertainty and lockdown difficulty were the most important for anxiety, and perceived threat was the most important for predicting lockdown adherence.

**Conclusions:**

During the extended lockdown, psychological symptoms increased, being enhanced by mental fatigue, cognitive difficulties and financial concerns. We found no evidence of behavioural fatigue. Thus, feeling mentally fatigued is not the same as being behaviourally fatigued.

## Background

Since the beginning of the COVID-19 pandemic, warnings have been issued about its impact on the mental health of affected populations. The experience of previous pandemics, such as the 2003 SARS and 2014–2015 Ebola outbreaks, indicated that fear of infection had spread vigorously in populations at risk, resulting in elevated levels of health anxiety.^1,2^ In addition, lockdowns have been shown to provoke negative psychological effects, including stress, low mood, irritability, anger and post-traumatic stress symptoms.^3^ Reports from countries first affected by COVID-19 confirmed these presumptions, revealing a significant impact on mental health symptoms.^4–7^

Similarly, in a former study carried out by our group during the first week of the national lockdown in Argentina, we found early affective symptoms similar to those reported in other countries.^8^ Participants evidenced elevated levels of anxiety and depressive symptoms associated with feelings of loneliness, daily stress and repetitive negative thinking as the main explanatory variables. Increases in health anxiety and behavioural effects of social isolation, reduction in levels of activity, diminished reward and disrupted routines were invoked as potential mechanisms to explain the observed emotional outcomes.^8^ However, differing from previous reports, our observations occurred when there were few cases and deaths in the country. Strong, generalised and sustained protective measures were adopted early in the outbreak in Argentina, compared with other nations, as the main sanitary strategy to reduce the spread of the virus in the population. By 20 March 2020, when the national lockdown began in Argentina, there were 31 cases and zero deaths in the country.^9^ This decision carried a prominent consequence: a large-scale preventive lockdown that was extended over several months, alongside the progressive growth of the pandemic. Such a prolonged lockdown created new questions about its effects on the confined population beyond its initial impact.

## Aims and hypotheses

This study aimed to explore, first, if the prolonged lockdown was associated with elevated symptoms of anxiety and depression; second, if accumulated mental fatigue was associated with adherence to lockdown, a phenomenon called ‘behavioural fatigue’; and third, if financial concerns were associated with adherence to lockdown and psychological symptoms.

Regarding the first question, we intended to explore if the persistence of the stressor was accompanied by a persistence of psychological symptoms, specifically, anxiety and depression symptoms. It has been suggested that the more prolonged the confinement, the more deleterious its consequences.^3^ However, a longitudinal study in the UK showed that the highest levels of depression and anxiety occurred in the early stages of lockdown, but declined quickly, as most individuals adapted to the circumstances.^10^ At the same time, other studies have suggested that as the pandemic spreads and the number of strains rises, the negative emotional correlates also increase.^11^ Particularly, in Argentina, the number of new contagion strains augmented slowly but steadily, with the continuous lockdown in place. A paradoxical situation was created in which, despite robust measures, infections continued to increase. We hypothesised that as long as the stressors persisted, psychological symptoms would remain stable or increase. In addition, as shown in previous studies, several factors, such as feelings of loneliness, intolerance of uncertainty, negative thinking and daily stress, were expected to be associated with the levels of psychological symptoms.^8,12–16^

Regarding the second question, the prolonged lockdown in Argentina created conditions to explore the controversial issue of behavioural fatigue, whereby individuals begin to ignore the regulations they once followed despite the ongoing risk. Discussions about the concept of behavioural fatigue arose when governments initiated restrictions in response to the first wave of COVID-19 in Europe. Public officials and experts in the UK warned about the possible negative effects of establishing a premature lockdown. It was assumed that if the countermeasures were too strict and premature, affected people could develop behavioural fatigue, with the undesirable consequence of eroding adherence with sanitary protective measures in general. This idea was heavily criticised by behavioural scientists who stated that the prediction lacked empirical support.^17,18^ However, as far as we know, no study has reported measures for behavioural fatigue in the general population during the COVID-19 pandemic, or tested its role as a factor in non-adherence to protective measures. In the present study, we assessed mental fatigue with a specific questionnaire together with self-reported adherence to lockdown, assuming as the main hypothesis that if behavioural fatigue occurred, then these two variables should be negatively correlated. As a secondary hypothesis, we expected that mental fatigue would be positively correlated with psychological symptoms.

Our third question concerned the role of the socioeconomic situation in Argentina, as a low- and middle-income country struggling with consecutive economic crises, high prevalence rates of poverty and many people working in informal positions. The suspension of work and economic activities seems to have created additional stressful life conditions and increasing financial concerns. Consequently, we wanted to evaluate how income and financial concerns interacted with psychological symptoms and attitudes toward lockdown. As hypotheses, we expected that financial concerns would be positively correlated with psychological symptoms, and negatively correlated with lockdown adherence.

Finally, we explored through regression models which factors in conjoint predicted our three main target variables: depression, anxiety, and lockdown adherence.

## Method

### Participants

This report is based on a sample of 3617 individuals from Argentina who were aged >18 years. Gender was reduced to three main categories: female, male and other. The education profile was segmented into four categories, in line with the national education system (see Table 1 for details). The family's basic income was asked in monthly Argentine pesos and converted to three categories (low, medium and high income). Because of considerable differences in COVID-19 contagion rate, the sample was divided into two groups ‘AMBA’ (Buenos Aires city and Great Buenos Aires with higher rates) and ‘non-AMBA’ (for the rest of the country). All participants gave their informed consent, following the Declaration of Helsinki and national laws, by clicking on the first screen of the survey platform, in which the objectives, characteristics, and risks of the study were explained. Only the participants who accepted the terms of consent were able to proceed to the subsequent questions, and the agreement was recorded in the survey database. The procedure of informed consent and the study were approved by the Ethics Committee of Favaloro Foundation (registration code: 1257).

**Table 1:** Sociodemographic and COVID-19-related data

	Total sample, % (*n* = 3617)	18–25 years, %	26–44 years, %	45–64 years, %	≥65 years, %
Gender					
Female	85.2	79.3	84.8	86.6	82.9
Male	14.5	19.3	14.8	13.3	17.1
Other	0.3	1.5	0.4	0.1	0.0
Education					
7–12 years	2.9	0.7	1.8	3.2	7.2
12–14 years	35.9	20.7	32.4	40.4	35.1
15–19 years	52.6	78.5	58.1	46.5	47.5
20+ years	8.7	0.0	7.8	9.8	10.2
Family income					
Low	39.3	47.4	41.1	35.9	43.9
Medium	37.4	37.6	36.6	38.4	35.4
High	23.4	15.0	22.4	25.7	20.8
Perception of COVID-19 threat					
Very low	12.0	29.6	14.0	9.6	8.3
Low	21.8	27.4	25.9	20.2	9.7
Medium	24.6	25.9	26.0	24.4	18.8
High	41.7	17.0	34.1	45.8	63.3
Perceived personal risk of infection					
Very low	22.5	34.1	23.3	21.6	19.3
Low	30.6	33.3	31.5	31.4	22.5
Medium	23.8	16.3	23.7	23.9	27.1
High	23.0	16.3	21.5	23.1	30.9
Adherence to lockdown					
Yes	79.7	76.3	80.3	78.3	85.1
No	2.8	4.4	2.7	2.7	3.0
Partially	17.5	19.3	17.0	19.0	11.9

### Contextual measures: stringency and mobility

We provide stringency and mobility data to show how Argentina ranks compared with other countries, regarding lockdown measures.

#### Stringency

Data were obtained from the Oxford COVID-19 Government Response Tracker,^19^ which tracks school and workplace closures, public gatherings, travel bans and stay-at-home exceptions, among other indicators. Measures were obtained at the mid-point of data collection (28 May 2020), after 70 days of lockdown.

#### Mobility

For a general overview of how mobility decreased during quarantine, we used data from Google Community Mobility Reports (google.com/covid19/mobility) from users who had turned on the location history setting. This index is then smoothed to a rolling 7-day average, and we grouped the subregions into the Metropolitan Area of Buenos Aires and the rest of the country.

### Instruments

The survey included two standardised questionnaires to assess the severity of depressive and anxiety symptoms, a questionnaire to evaluate mental fatigue and several additional measures to assess related variables. Instruments are presented in the order in which they were administered.

#### Sociodemographic characteristics

Potentially relevant general characteristics, such as age, gender, family income, region of the country and level of education, were surveyed.

#### Perception of COVID-19 and attitudes toward lockdown

The survey included questions created *ad hoc* to evaluate variables related to the pandemic and lockdown. Perception of the threat of COVID-19 (severity of its outcomes) and perception of the risk of being infected (susceptibility) were explored as two single dimensions, on a scale from 0 (‘not at all’) to 10 (‘extreme’). Attitudes toward lockdown were evaluated by two self-reported dimensions: adherence with the measure (from 0 ‘not at all’ to 10 ‘completely adherent’) and subjective difficulty of complying with the lockdown (from 0 ‘not at all’ to 10 ‘absolutely difficult’).

#### Financial concerns

Present and future financial concerns were explored through two specific single-dimension questions, from 0 ‘desperate (can't afford essentials)’ to 10 ‘not worried at all’. Present worries correspond to the financial situation at the time of the survey, whereas future worries correspond to an estimation of the financial situation 12 months later. In both cases, a lower rating indicated a more negative evaluation.

#### Daily stress

Impact on daily life was assessed through an index that evaluates perceived stress within five domains: work, household chores, physical exercise, leisure activities with children, and relationships with other adults. The questionnaire is described in our previous report.^8^

#### Patient Health Questionnaire-9

The Patient Health Questionnaire-9 (PHQ-9) is a brief self-report scale composed of nine items based on the DSM-IV criteria for the diagnosis of major depressive episodes. The Argentine version of the PHQ-9^20^ has high internal consistency (Cronbach's alpha of 0.87) and satisfactory convergent validity with the Beck Depression Inventory-II scale (Pearson's *r* = 0.88, *P* < 0.01). The cut-off points established by Urtasun et al^20^ were used to evaluate the possible diagnosis of depression and the range of severity in the present study. A score of ≥8 indicated a possible diagnosis of major depression according to the DSM-IV. The cut-off points for severity ranges were 6–8 for mild symptoms, 9–14 for moderate symptoms and ≥15 for severe depressive symptoms.

#### Generalized Anxiety Disorder-7

The Generalized Anxiety Disorder-7 (GAD-7) is a brief seven-item self-report questionnaire designed to identify probable cases of generalised anxiety disorder and assess the severity of symptoms^21,22^ The Spanish for Argentina version of the GAD-7 was used in this case (downloaded from: https://www.phqscreeners.com/select-screener). The GAD-7 was utilised in previous studies in Argentina,^23^ and showed a high internal consistency for the present study (Cronbach's alpha of 0.90). To establish the severity levels of the current sample, the cut-off points from Spitzer et al^22^ were used. A score of ≥10 was considered as indicative of the presence of a possible anxiety disorder.

#### Intolerance of Uncertainty Scale – 12-item

Intolerance of uncertainty is defined as a ‘dispositional characteristic that reflects a set of negative beliefs about uncertainty and its implications’.^24^ People with difficulties tolerating uncertainty tend to believe that uncertainty in itself is distressing, unfair and should be avoided.^25^ The Intolerance of Uncertainty Scale – 12-item is a short version of the original 27-item Intolerance of Uncertainty Scale.^26^ The Intolerance of Uncertainty Scale has been adapted and psychometrically validated in Argentina.^27^ Cronbach's alpha for our sample was very high (0.92).

#### Negative repetitive thinking

Individuals with emotional disorders usually report excessive and repetitive thinking about their current concerns, problems and past experiences, and worries about the future.^28^ In the current study, we explored this dimension by assessing the presence of an increased number of negative thoughts related to past, future or interpersonal concerns since the beginning of the lockdown. For each of these options, there was a categorical (yes/no) answer. Negative repetitive thinking was considered present when at least one of the options was selected.

#### Current mental health treatment

Being in current psychotherapeutic or psychopharmacological treatment for a previous mental health condition was asked through an *ad hoc* question as a proxy for a potential pre-existing disorder.

#### Mental fatigue

This term refers to the feeling that people may experience after or during prolonged periods of cognitive activity.^29^ For our study, we employed the five items measuring mental fatigue (items 3 and 6–9) from the Fatigue Assessment Scale-10.^30^ This scale asks respondents how they usually feel on a rating scale from 1 (never) to 5 (always). The original instrument showed an adequate internal consistency of 0.87. Cronbach's alpha for our sample for the mental fatigue subscale was 0.82.

#### Cognitive failures

This dimension was evaluated with an *ad hoc* questionnaire that included seven single-dimension questions exploring self-perceived difficulties in attention, planning, memory and decision-making in a real-life context. Each question was rated on a Likert scale with five options, from ‘totally agree’ to ‘totally disagree’. The response was interpreted as positive when participants chose ‘agree’ or ‘totally agree’. The final index of cognitive failures was the sum of the seven item responses, ranging from 0 to 7.

#### Feelings of loneliness

Loneliness was measured with the UCLA Loneliness Scale (UCLA-LS^31^), adapted to Argentine Spanish by Sacchi and Richaud de Minzi.^32^ This 20-item Likert-type scale with four options (never, rarely, sometimes, often) asked about self-perception of social connections and negative feelings associated with loneliness as a unidimensional construct. Cronbach's alpha for our sample was 0.91.

### Procedures

The link to the online survey was distributed through different social networks (Facebook, Twitter, Instagram, WhatsApp) and email. The questionnaire went online on 21 May 2020 and the recruitment of the present sample was completed in 15 days. The official start of the national lockdown in Argentina was established at 12 am on 20 March 2020, so the responses obtained correspond to a period of between 63 and 77 days of restriction (mean 72, s.d. 4.02).

### Statistical analysis

Comparisons of continuous variables (depression, anxiety, mental fatigue, loneliness, risk and threat perception, lockdown adherence and financial concerns) between different sociodemographic subgroups (by age, gender, income, region and current mental health treatment) were made by one-way ANOVA followed by Tukey's honestly significant difference test or Tamhane's T2 test for *post hoc* comparisons, where appropriate. Correlations between measures were carried out with the Pearson correlation coefficient, with Bonferroni correction for multiple comparisons. When analysing categorical variables, the Pearson *χ*^2^-test was used.

Least absolute shrinkage and selection operator (LASSO) regression was used through the *sklearn* package, to develop predictive machine-learning models for the three main target variables (depression, anxiety and lockdown adherence). We used an 80:20 train:test split. Data was standardised and the L1 penalty coefficient was selected through ten-fold cross-validation on the training set by testing 30 equally spaced values on a log scale from 0.0001 to 1. Then, the optimal penalty coefficient was used to train the model and predict the test set. All variables from the pairwise correlation analysis were included in the regression model together with additional independent variables, such as negative repetitive thinking (ordinal), family income (ordinal) and gender (categorical; converted to two dummy variables for male and female as there were only nine samples of different gender identity).

## Results

### Sociodemographic characteristics

The sample size was 3617 individuals. The mean age of the participants was 47.31 years (s.d. 12.76). Female gender was most commonly reported (85.2%), and few respondents reported as a nonbinary gender identity or preferred not to answer (0.3%). There were participants from all of the country's provinces. Approximately half of the sample (*n* = 1765, 48.9%) were from the AMBA, where 37% of the country's inhabitants live. By the first day of the survey, 75.6% of the cases of COVID-19 in Argentina were concentrated in the AMBA. No age differences were found when comparing the AMBA (mean 49.11, s.d. 13.45) with the rest of the country (mean 45.59, s.d. 11.81). The sample was well-distributed across income levels. Regarding education, although all ranks are represented, there is a tendency toward overrepresenting higher education levels (Table 1).

### Contextual analysis: quarantine, stringency and mobility

Argentina ranks 14th out of 184 countries on the mean stringency index since the start of data collection (1 January 2020) and fifth on the number of continuous days with stringent quarantine of 85/100 or more (see Supplementary Table 1 available at https://doi.org/10.1192/bjo.2021.1065 and Supplementary Figure 1 for the trends of quarantine stringency increased and oscillated worldwide).

Mobility decreased by 20%, to almost 100% during quarantine, whereas time at home increased by up to 40% compared with baseline (15 February 2020) (Supplementary Figure 2). The order of the largest decreases in mobility was: parks, retail and recreation, transit stations, workplaces, and grocery and pharmacy. Mobility changes were similar between the AMBA and the rest of the country. We additionally included measures of infection rate and death rate by COVID-19. At the beginning of the survey, there were 9931 cases of COVID-19 in the country and 433 deaths. On final day of the survey, cases had increased to 20 197 and deaths had increased to 608;^9^ therefore, the death rate was quite low.

### Perception of COVID-19 and adherence to lockdown

Regarding the perception of COVID-19 threat (Table 1), most participants selected high (41.7%) and medium (24.6%) ratings, whereas low (21.8%) and very low (12%) were less frequent. Segmented by age, an opposite pattern was found between the youngest and oldest participants. Perception of COVID-19 threat was rated as very low by 29.6% of participants aged 18–25 years, and only 17% of this age group perceived a high threat. In contrast, 63.3% of older adults (≥65 years) rated the threat as very high, and only 8.3% estimated a very low threat. Divided by territory, participants from AMBA showed a higher threat perception than the rest of the country (*F*(1,3615) = 25.16, *P* < 0.001, *η*^2^ = 0.007).

For perceived risk of infection, 30.6% rated their risk of infection as low, 23.8% as medium, 23% as high and 22.5% as very low risk. Segmented by age, the results presented a similar trend to perceived threat, with older adults perceiving a higher risk of infection than younger adults, but with smaller differences (Table 1).

Divided by area, participants from AMBA showed a higher perception of risk of being infected than those from the rest of the country (*F*(1,3615) = 34.64, *P* < 0.001, *η*^2^ = 0.009).

Most participants perceived themselves as adherent to lockdown procedures (79.7) (Table 1). Partial adherence was higher in the younger group (19.3%) and lower in the older group (11.9%) (Table 1). Women were more adherent than men (*F*(1,3606) = 31.27, *P* < 0.001, *η*^2^ = 0.01), and participants from AMBA were more adherent than participants from the rest of the country (*F*(1,3615) = 8.41, *P* = 0.004, *η*^2^ = 0.002). Lockdown adherence was correlated with perceived threat of COVID-19 (*r* = 0.249, *P* < 0.0001) and perceived risk of infection (*r* = 0.171, *P* < 0.0001). A total of 41.4% of the total sample experienced the lockdown as highly difficult, whereas 24.5% and 34.1% of the sample reported medium and low ratings of perceived difficulty, respectively.

### Mental health symptoms

#### Depression symptoms

Analysis of PHQ-9 score revealed that 45.6% of the sample surpassed the cut-off score for a possible diagnosis of depression. Severity ranges are presented in Table 2. When divided by age, the younger group (18–25 years) was the most depressed, with 64.4% of participants reporting moderate and severe scores, followed by 40.1% for those aged 25–44 years, 35.3% for those aged 45–64 years and 28.5% for those aged ≥65 years (Table 2).

**Table 2:** Psychological symptoms

	Total sample	Age range, years			
		18–25	25–44	45–64	≥65
Depression scores (PHQ-9)					
Mean (s.d.)	8.18 (5.84)	11.34 (5.73)	8.60 (5.75)	7.81 (5.85)	6.91 (5.59)
Depression severity (PHQ-9), %					
None	37.8	14.1	34.0	41.0	48.1
Mild	24.5	21.5	25.9	23.7	23.5
Moderate	22.3	37.0	23.8	21.3	15.2
Severe	15.4	27.4	16.3	14.0	13.3
Percentage of the sample surpassing cut-off for depression (PHQ-9)					
Not depressed	54.4	25.9	51.1	57.9	63.0
Possibly depressed	45.6	74.1	48.9	42.1	37.0
Anxiety scores (GAD-7)					
Mean (s.d.)	7.03 (5.17)	8.38 (4.94)	7.52 (5.21)	6.74 (5.16)	5.84 (4.83)
Anxiety severity (GAD-7), %					
None	46.7	33.3	42.5	56.6	46.7
Mild	26.3	28.1	26.9	24.6	26.3
Moderate	16.5	27.4	18.8	11.0	16.5
Severe	10.5	11.1	11.9	7.7	10.5
Percentage of the sample surpassing cut-off for anxiety (GAD-7)					
Not anxious	73	61.5	69.4	75.4	81.2
Possibly anxious	27	38.5	30.6	24.6	18.8
Loneliness Scale, %					
Low	66.3	43.0	66.4	68.0	67.1
Medium	17.1	28.1	18.1	16.0	14.4
High	10.4	20.0	10.3	9.6	10.2
Extreme	6.2	8.9	5.2	6.3	8.3

PHQ-9, Patient Health Questionnaire-9; GAD-7, Generalized Anxiety Disorder-7.

Between-groups comparison (one-way ANOVA) showed that participants in current mental health treatment had significantly higher levels of depression (*F*(1,3615) = 103.56, *P* < 0.001, *η*^2^ = 0.028). However, participants without current treatment also exhibited elevated rates of depressive symptoms (42.2% scoring above the cut-off and 34.3% with moderate or severe symptoms).

There were also differences in depression scores between age groups (*F*(3,3613) = 24.29, *P* < 0.001, *η*^2^ = 0.025). *Post hoc* comparisons showed differences between all groups (18–25 > 26–45 > 46–64 > 65+) showing that depression scores decreased with age. Also, women were significantly more depressed than men (*F*(2,3614) = 14.13, *P* < 0.001, *η*^2^ = 0.008). Finally, significant differences in depression scores according to income were found (*F*(1,3615) = 50.57, *P* < 0.001, *η*^2^ = 0.013). *Post hoc* comparisons revealed differences between the three groups (low > medium > high; *P* < 0.001). There were no differences in depression scores between AMBA and the rest of the country (*P* < 0.14).

#### Anxiety severity

Global ratings of the GAD-7 showed that 46.7% of participants scored minimal or no anxiety symptoms, followed by 42.8% with mild and moderate symptoms and 10.5% with severe symptoms (Table 2). A possible diagnosis for an anxiety disorder (>10) was found in 27% of the total sample. Thus, ratings of anxiety also support our hypothesis concerning the persistence of emotional symptoms during the extended lockdown.

Divided into age subgroups, the 18–25 years and 25–44 years groups displayed higher rates of anxiety (Table 2). As in the case for depression, being on current treatment for a mental health condition (*F*(1,3615) = 144.79, *P* < 0.001, *η*^2^ = 0.039) and being a woman (*F*(2,3614) = 11.42, *P* < 0.001, *η*^2^ = 0.006) were associated with higher levels of anxiety. Comparison between income groups revealed significant differences in anxiety scores (*F*(2,3614) = 16.76, *P* < 0.001, *η*^2^ = 0.009). *Post hoc* comparisons revealed differences between the three groups (low > medium = high; *P* < 0.001); namely, that participants with low incomes have higher anxiety scores than both medium and high income groups, while there were no differences between medium and high income groups. There were no group differences in anxiety scores between AMBA and the rest of the country (*P* = 0.52).

#### Correlates of psychological symptoms

A total of 38.1% of the sample endorsed a negative global daily life stress index. There were no differences in daily life stress between AMBA and the rest of the country (*P* = 0.42). Daily life stress was correlated with depression (*r* = −0.306, *P* < 0.0001) and anxiety scores (*r* = −0.292, *P* < 0.0001) (Table 3).

**Table 3:** Correlations between quantitative variables (Pearson's correlation coefficients)

	1	2	3	4	5	6	7	8	9	10	11	12	13
Depression (PHQ-9)	1												
Anxiety (GAD-7)	0.743*	1											
Mental fatigue	0.644*	0.525*	1										
Cognitive failures	0.549*	0.491*	0.495*	1									
Intolerance of uncertainty	0.436*	0.478*	0.384*	0.326*	1								
Loneliness	0.520*	0.481*	0.467*	0.446*	0.341*	1							
Daily stress	−0.306*	−0.292*	−0.247*	−0.192*	−0.177*	−0.239*	1						
Age	−0.124*	−0.107*	−0.149*	−0.008	−0.063*	−0.034	0.050	1					
Perceived threat	0.023	0.040	0.005	0.054	0.053*	0.039	0.01	0.228*	1				
Perceived risk	0.068*	0.092*	0.044	0.068*	0.104*	0.056	−040	0.088*	0.561*	1			
Lockdown adherence	−0.060*	−0.049*	−0.059*	−0.060*	0.028	−0.042*	0.043	0.038	0.249*	0.171*	1		
Lockdown difficulty	0.330*	0.356*	0.224*	0.246*	0.153*	0.251*	−0.265*	−0.025	−0.023	0.028	−0.051	1	
Financial concerns (present)	−0.237*	−0.228*	−0.155*	−0.149*	−0.084*	−0.168*	0.022	0.048	0.012	0.003	0.101	−0.129*	1
Financial concerns (future)	−0.262*	−0.262*	−0.203*	−0.185*	−0.126*	−0.178*	0.081*	0.072*	−0.001	−0.036	0.077	−0.158*	0.732*

PHQ-9, Patient Health Questionnaire-9; GAD-7, Generalized Anxiety Disorder-7.

* *P* < 0.0001 and are significant after Bonferroni correction for multiple comparisons (corrected *α* = 0.0003).

Regarding subjective loneliness, 17.1% reported medium and 16.6% high ratings on the UCLA-LS. More intense feelings of loneliness appeared in the group aged 18–25 years (28.9% in high and extreme grades). AMBA showed significant lower levels of loneliness (*F*(1,3615) = 4.829, *P* = 0.028, *η*^2^ = 0.001) than the rest of the country. Loneliness was positively correlated with scores of depression (*r* = 0.52, *P* < 0.01) and anxiety (*r* = 0.48, *P* < 0.01) (Table 3).

A total of 73.6% of the total sample expressed at least one kind of negative repetitive thinking during the period of lockdown, with higher rates for younger participants (87.4%). The group with negative repetitive thinking had significantly higher depressive (*F*(1,3615) = 481.05, *P* < 0.001, *η*^2^ = 0.117) and anxiety scores (*F*(1,3615) = 521.77, *P* < 0.001, η^2^ = 0.126) than the group without negative repetitive thinking.

Finally, intolerance of uncertainty was positively associated with both depression and anxiety (*r* = 0.436, *P* < 0.0001 and *r* = 0.478, *P* < 0.0001, respectively).

### Mental fatigue and cognitive failures

Contrary to the main hypothesis of behavioural fatigue, mental fatigue showed a significant but negligible negative correlation with adherence to lockdown (*r* = −0.059, *P* < 0.0001; Table 3). However, mental fatigue was positively correlated with depression (*r* = 0.644, *P* < 0.0001) and anxiety (*r* = 0.525, *P* < 0.0001), supporting our secondary hypothesis. Even if mental fatigue was not associated with adherence, it was positively correlated with lockdown difficulty (*r* = 0.224, *P* < 0.0001). There was no difference in mental fatigue between AMBA and non-AMBA participants (*P* = 0.30).

Cognitive failures were positively correlated with depression (*r* = 0.549, *P* < 0.0001), anxiety (*r* = 0.491, *P* < 0.0001) and mental fatigue (*r* = 0.495, *P* < 0.0001). Additionally, 62.48% of the sample experienced failure in three or more cognitive areas.

### Financial concerns

As expected, both present and future financial concerns were associated with depression, anxiety, mental fatigue, cognitive failures and loneliness (Table 3). In contrast, financial concerns were not correlated with adherence to lockdown measures. There were differences in present and future worries between the three income groups (*F*(2,3614) = 180.32, *P* < 0.001, *η*^2^ = 0.091; low > medium > high) showing that worries decreased as income increased.

### Predictive models of depression, anxiety and lockdown adherence

Three LASSO regression analyses were carried out to evaluate the predictive role of the different variables over depression, anxiety and lockdown adherence (Table 4).

**Table 4:** Variable importance when predicting outcome variables with LASSO regression

	Depression (*R*^2^ = 0.69)		Anxiety (*R*^2^ = 0.63)		Lockdown adherence (*R*^2^ = 0.09)	
Importance	Covariate	Coefficient	Covariate	Coefficient	Covariate	Coefficient
1	GAD-7	2.57	PHQ-9	2.62	Perceived threat	0.39
2	Mental fatigue	1.53	Intolerance of uncertainty	0.83	Female	0.12
3	Cognitive failures	0.68	Lockdown difficulty	0.48	Financial concerns (present)	0.1
4	Loneliness	0.49	Cognitive failures	0.32	Perceived risk	0.09
5	Daily stress	−0.29	Loneliness	0.31	Negative thinking	−0.08
6	Lockdown difficulty	0.23	Family income	0.29	Intolerance of uncertainty	0.08
7	Financial concerns (present)	−0.17	Negative thinking	0.29	Cognitive failures	−0.07
8	Family income	−0.16	Daily stress	−0.21	Daily stress	0.05
9	Intolerance of uncertainty	0.14	Financial concerns (present)	−0.19	Family income	0.05
10	Age	−0.12	Financial concerns (future)	−0.19	GAD-7	−0.05
11	Female	0.1	Perceived risk	0.13	Age	−0.02
12	Negative thinking	0.09	Female	0.12	PHQ-9	−0.02
13	Financial concerns (future)	−0.02	Mental fatigue	0.1	Mental fatigue	0
14	Lockdown adherence	0	Age	−0.07	Lockdown difficulty	0
15	Male	0	Lockdown adherence	−0.06	Financial concerns (future)	0
16	Perceived threat	0	Perceived threat	0.01	Loneliness	0
17	Perceived risk	0	Male	0	Male	0

The standardised coefficients represent how many standard deviations a dependent variable will change per standard deviation increase in the covariate (e.g., 1 s.d. increase in mental fatigue predicts 1.53 increase in PHQ-9, if all other covariates are fixed). More important features have higher absolute coefficient values. Increases in covariates with positive coefficients make it more likely that the dependent variable will increase, and those with negative coefficients make it more likely that the dependent variable will decrease. Coefficients closer to zero are not important. LASSO, least absolute shrinkage and selection operator; GAD-7, Generalized Anxiety Disorder-7. PHQ-9, Patient Health Questionnaire-9.

The covariates explained 69% of the variance when predicting depression (PHQ-9 total score), and the most important variables, in order, were anxiety (GAD-7 total score), mental fatigue, cognitive failures, loneliness, family income, daily stress and lockdown difficulty. When predicting anxiety (GAD-7), the covariates explained 63% of the variance, and the most important variables were depression (PHQ-9), intolerance to uncertainty, lockdown difficulty, cognitive failures, loneliness, family income and negative thinking. Finally, the covariates explained only 9% of the variance of lockdown adherence predictions, and the most important variable was the perceived threat.

## Discussion

This study aimed to evaluate the emotional and cognitive correlates of the COVID-19 pandemic alongside a lockdown extended between 63 and 77 days in Argentina. Regarding the first question, as hypothesised, ratings of depression and anxiety were higher than those observed in a previous report 2 months before (45.2 *v*. 33.7% for depression, and 27 *v*. 23.2% for anxiety^8^). We interpret these findings as an indication that the emotional state of the population accompanied the negative trajectory of the pandemic and the increasing rate of infection. In agreement with a former report, previous mental health treatment, low income, younger age and being female were associated with higher levels of depression and anxiety in the present study. Also, the obtained figures are higher than expected, based on pre-pandemic studies. A national representative community survey carried out in 2018 in Argentina found a 12-month prevalence of 5.7% for any mood disorder and 9.4% for any anxiety disorder.^33^ Another study in two cities of Argentina in 2017 that used the PHQ-9 found a prevalence of major depressive episodes of 5.6% and 9.5%, respectively.^34^ However, it should be noted that self-report methods may overestimate the rate of psychiatric disorders.

Regarding the second question, the issue of behavioural fatigue, we observed that mental fatigue was highly correlated with emotional symptoms, and was the second most important variable for predicting depression after anxiety symptoms. Also, after an average of 72 days of confinement, mental fatigue was associated with perceiving the lockdown as a difficult experience. Notwithstanding, in contradiction with the hypothesis about the behavioural effects of mental fatigue, most people were highly adherent to the imposed restrictions. The main effect of mental fatigue was manifest at the emotional level instead of the behavioural level, at least in the 63–77 days covered by the present study. According to the present findings, feeling mentally fatigued is not the same as being behaviourally fatigued.

Regarding our third question about financial concerns, although it may be plausible that worrying about the economy would be associated with relaxing lockdown adherence, we did not find this effect: financial concerns were not associated with lockdown adherence at the time of the present study. As an explanation for this failed prediction, we can speculate that the expected negative effect of financial concerns on lockdown adherence may have been tempered by the significant financial assistance provided by the national government to individuals and companies with difficulties in maintaining their work activities during the first months of the pandemic. In contrast, financial concerns were moderately correlated with emotional symptoms, and were not important variables when taking into account other covariates in the regression models. However, family income appears to contribute to the prediction of anxiety, albeit slightly.

Together, these findings indicate that prolonged restrictions have an emotional cost, but people may feel compelled to maintain safety behaviours for different reasons, despite fatigue and financial concerns. In our study, only the perceived threat of COVID-19 and perceived risk of infection correlated with lockdown adherence. This fact, alongside the significant levels of anxiety registered in the population, may imply that threat appraisal is the main relevant driver for people to adopt protective measures. High anxiety scores may suggest that people were still worried about the threat of COVID-19 as the number of cases continued to increase at the time of the survey, and this fear could have reinforced lockdown adherence. The perceived threat (severity of the disease) and the risk of infection (susceptibility) could be understood as the cognitive components of the appraisal, whereas anxiety reflects its emotional correlate.^35^ Consistent with these findings, before the COVID-19 outbreak, a review by Bish and Michie^36^ on the determinants of protective behaviours during pandemics found that higher levels of perceived susceptibility and perceived severity of disease, as well as higher state anxiety levels, are associated with preventive behaviours.

Although mental fatigue did not appear to be related to lockdown adherence, it was found to be the second most important factor predicting depression in the regression model. In the context of the pandemic, mental fatigue can result from prolonged cognitive efforts to maintain safety behaviours and sustained inhibitory control in restricting habitual behaviours, such as close physical contact. Moreover, the hypervigilance associated with anxiety and fuelled by the incessant flow of threatening news can lead to mental fatigue. Cognitive failures, another factor associated with depression, may be linked to mental fatigue, since research shows that fatigued individuals have difficulties in focusing their attention, planning and adaptively changing strategies in the face of negative outcomes.^29^ In addition, as we observed in our previous report,^8^ the feeling of loneliness was another important factor related to depression, possibly magnified by prolonged social restrictions for specific groups at risk.^37^ In the case of anxiety, the regression model supported the significant role that intolerance of uncertainty plays in generalised anxiety and worry, according to cognitive–behavioural models.^24,25,38^

Interestingly, there were no differences in psychological symptoms and mental fatigue between AMBA and the rest of the country, even if the former region was much more affected by COVID-19. A possible explanation entails the exposure to national news and social media that may have activated negative emotions in advance.^39^

As we face new waves of COVID-19, this study has several potential practical implications. First, there is no conclusive evidence in our study for supporting the phenomenon of behavioural fatigue after an average of 72 days of stringent lockdown. Despite the high levels of fatigue and emotional symptoms, most people strongly adhered to the established lockdown. A plausible interpretation of this fact is that perceived threat and perceived risk of infection were the main drivers of the high adherence to lockdown measures despite its negative emotional consequences. Therefore, sustained societal awareness of the persistence of the perils of the pandemic could constitute a relevant factor to maintaining protective behaviour or initiating new restrictive measures. At the same time, a strong fear-promoting communication focusing on negative outcomes and threats may worsen the emotional status of the population. As noted by Bish and Michie,^36^ communications designed to highlight perceptions of risk should also be combined with advice as to how the threat can be alleviated. Furthermore, since mental fatigue may be indicating that people have a negative subjective experience of lockdown despite being adherent, it may be helpful to communicate clear examples and simple figures of how effort invested leads to positive results.

A second practical implication is that public health strategies should be considered to ensure adequate coverage of the wide range of unprecedented mental health necessities created by the pandemic. These include the provision of mental health treatment to individuals with pre-existing conditions, identifying and assisting vulnerable groups, allowing rapid detection and access to care for new emerging cases, and promoting preventive behaviours oriented to the wellness of the general population.

### Limitations

The present study has several limitations. First, the survey was disseminated incidentally. Nevertheless, all of the country's provinces were sampled. In addition, as the survey was disseminated through social media networks and email, it is possible that a bias occurred toward participants with higher education and income levels. Second, as noted previously, self-report methods may overestimate the rate of psychiatric disorders in comparison with the more reliable gold standard of diagnostic interviews. A recent meta-analysis about the use of the PHQ-9 for the screening of major depressive episodes in primary care found that approximately half of patients with positive screens could be false positives.^40^ It is important to prudentially consider the present results and avoid jumping to clinical conclusions. Complementary and more precise procedures should be adopted to confirm or reject any assumption of diagnosis. Third, our sample was unbalanced in gender, with women being overrepresented. Female gender is associated with increased rates of anxiety and depression in epidemiological studies, so sampling bias may have inflated the figures of emotional symptoms in our study. Fourth, because of the observational nature of the study, it is not possible to disentangle the effects produced by the pandemic itself from the impact of the lockdown. Our analyses are not intended to estimate the causal effect between variables or be an exhaustive analysis of the context (e.g. quarantine, stringency, mobility, death rate). The contextual analysis provides quantitative evidence that, at the time of the study, Argentina had one of the most continuously stringent lockdowns with low mobility, and a low death rate. A more exhaustive analysis could include measures of unemployment, Gini index for income inequality, intensive care unit occupancy and stimulus packages, which could all help to explain symptoms; however, some of these measures are not yet available and are not the focus of the analysis. Similarly, we cannot separate economic concerns resulting from the effects of the pandemic and lockdown from pre-existing concerns about the country's economic situation, and both may likely have contributed to the concerns reflected in the survey. Fifth, the instruments were not presented in random order and, consequently, we cannot rule out that the sequence in which they were completed had spurious effects on the responses to subsequent questionnaires. Finally, since the sample of the present study is not the same as that in our previous report,^8^ the increment in anxiety and depression scores between the two time points may be a result of sampling differences or other potential confounding variables. However, the observed rates of symptoms in the current study are elevated enough to assert that psychological symptoms remained high during the lockdown, and that there was no habituation or adaptation to the situation.

In conclusion, psychological symptoms persisted during the extended lockdown, as evidenced by high levels of depression and anxiety symptoms, but we found no evidence of behavioural fatigue, as lockdown adherence remained high. Rather, mental fatigue, cognitive failures, intolerance of uncertainty, loneliness and financial concerns may be considered as components of the emotional impact of the pandemic.

## Data Availability

Code and data are openly available at github.com/danielmlow/covid19_argentina.

## Supplementary material

For supplementary material accompanying this paper visit http://dx.doi.org/10.1192/bjo.2021.1065. click here to view supplementary material
